# Gene Therapy for Dystrophic Epidermolysis Bullosa

**DOI:** 10.1111/exd.70339

**Published:** 2026-08-07

**Authors:** Cristina Has, Meropi Karakioulaki

**Affiliations:** ^1^ Department of Dermatology and Venereology, Faculty of Medicine and Medical Center University Hospital Freiburg Freiburg Germany

## Abstract

Dystrophic epidermolysis bullosa (DEB) is a rare, debilitating genodermatosis caused by loss‐of‐function variants in *COL7A1,* resulting in type VII collagen (C7) deficiency and defective anchoring fibrils, which are essential for dermal–epidermal adhesion. *COL7A1* is a large gene, and C7 is synthesized by both keratinocytes and fibroblasts, and extracellularly secreted, where it forms a supramolecular network that stabilizes the dermal–epidermal junction. Human genotype–phenotype correlations suggest that residual C7 expression (~10% of normal levels) may confer a milder disease phenotype, whereas complete absence results in generalized skin fragility with scarring and systemic complications. Thus, restoring C7 expression remains a central therapeutic objective. Since the discovery of *COL7A1*, thousands of pathogenic variants have been described, including amino acid substitutions in the triple‐helical domain that allow partial C7 function, whereas null alleles are associated with severe phenotypes. Gene‐ and cell‐based strategies have evolved to address the large *COL7A1* size and delivery challenges. Ex vivo gene/cell therapies (keratinocytes or fibroblasts engineered to express *COL7A1*) have demonstrated partial, wound‐specific C7 restoration but with durability limitations, as grafted cells may be outcompeted or lose transgene expression over time; select patients show sustained anchoring fibril formation and improved wound healing; however, long‐term outcomes remain variable. In vivo topical gene therapy has achieved clinically meaningful wound closure in DEB, illustrating a paradigm shift toward accessible and patient‐friendly therapeutic approaches, albeit with real‐world considerations on durability and wound recurrence. A multidisciplinary framework integrating EB expert centers, drug‐delivery pharmacy services, wound management, and patient‐support programs can further enhance treatment access and adherence. General factors such as nutritional status, infection and comorbidities also influence both wound healing and the response to gene therapy. Revertant mosaicism offers a natural gene‐therapy substrate: cultured epidermal autografts derived from revertant patches have yielded substantial, yet heterogeneous, epithelization, with outcomes contingent on revertant cell proportion and culture fidelity. Collectively, these advances highlight the emerging potential of gene‐ and cell‐based therapies to achieve durable disease modification in DEB.

## 1 Introduction

Dystrophic epidermolysis bullosa (DEB) is a rare devastating genetic skin disorder caused by deficiency of type VII collagen (C7), the major component of the anchoring fibrils [1, 2]. Since the discovery of the *COL7A1* gene back in the '90 [3, 4], few thousands of distinct pathogenic variants were reported. These variants and their biallelic combinations cause a broad spectrum of phenotypes with skin and mucosal fragility. Absence of C7 is associated with a distinct phenotype consisting of severe generalized blistering, scarring, mutilations and systemic complications [5]. Aminoacid substitutions, in particular glycine residues in the triple helical domain, as well as in frame splicing alterations or deletions/duplications, may allow the production of C7 and the assembly of structurally altered but partially functional anchoring fibrils, which are associated with a spectrum of milder phenotypes [2, 5, 6].

*COL7A1* is one of the largest human genes, spanning approximately 31 132 base pairs of genomic DNA and comprising 118 exons, and is transcribed into an mRNA of 9287 bases. C7 is synthesized by keratinocytes and fibroblasts as large procollagen VII (pro‐α1(VII)) chains of approximately 290–300 kDa, which contain a central triple‐helical collagenous domain flanked by the non‐collagenous NC‐1 and NC‐2 domains. Three pro‐α1(VII) chains assemble into triple‐helical monomers, which further associate into anti‐parallel dimers and subsequently into anchoring fibrils at the dermal–epidermal junction [7]. Processing of procollagen VII into mature collagen VII involves proteolytic removal of the C‐terminal propeptide (NC‐2). Besides being crucial for the anchorage of the epidermis to the dermis, C7 and its supramolecular network provide a structural scaffold for the superficial dermis and the herein residing extracellular matrix [8]. As an integral component of the extracellular matrix, C7 has a relatively long half‐life of approximately 1 month in murine skin [9]. The critical amount of C7 required for a relative stability of the skin remains under debate, as does the relative contribution of keratinocytes and fibroblasts. In the mouse model, 30% of normal C7 levels, as well as expression from either keratinocytes or fibroblasts alone, appeared sufficient to maintain stable dermal‐epidermal adhesion [10, 11]. In contrast, human genotype–phenotype correlations suggest that C7 levels of around 10% of normal may be sufficient to provide dermal‐epidermal stability and are typically associated with milder DEB phenotypes [12]. A human study further suggests that C7 should be provided by both keratinocytes and fibroblasts in order to rescue the skin fragility phenotype [13].

Developing a gene therapy to improve wound healing and restore cutaneous stability in DEB has been a long‐standing goal of generations of researchers [14, 15, 16, 17, 18, 19, 20, 21, 22, 23, 24]. Current strategies focus either on the addition of a functional *COL7A1* gene or on the precise correction of pathogenic variants through gene editing, most commonly performed ex vivo in patient‐derived keratinocytes or fibroblasts. However, the translation of these approaches into clinical practice has proven more complex and time‐consuming than initially anticipated because of both general challenges associated with gene therapy (e.g., safety, manufacturing costs and technological limitations) and DEB‐specific obstacles, including the large size of *COL7A1*, the need for efficient and sustained C7 expression and the long‐term persistence of genetically corrected cells. Additional challenges arise from the extensive cutaneous and mucosal surfaces requiring treatment and the continuous turnover of epidermal keratinocytes. As a result, gene therapy approaches developed to date have largely been restricted to the treatment of localized wound areas. Nevertheless, the recent approval of two gene therapies marks a major milestone in the treatment of DEB. The aim of this review is to provide clinicians involved in the care of patients with DEB with a comprehensive overview of gene therapies that are currently approved or under clinical investigation, highlighting their mechanisms of action, clinical evidence, advantages, limitations and potential future role in patient management. For gene therapy approaches in pre‐clinical development we refer to recent review articles [25, 26, 27].

## 2 Approved Gene Therapies

### 2.1 Ex Vivo Cell and Gene Therapy

Ex vivo cell and gene therapy approaches for DEB have been developed over the past decades mainly by researchers at Stanford University [19, 20, 21]. In brief, patient‐derived keratinocytes are obtained from skin biopsies, transduced with a retroviral vector carrying the full‐length *COL7A1*, expanded in epidermal sheets, and subsequently transplanted on the wound surfaces. There is no enrichment for epidermal stem cells. This may have contributed to the limited long‐term persistence of transgene‐expressing cells with this approach, in contrast to the durable gene‐corrected epidermal autografts achieved in junctional epidermolysis bullosa [28, 29, 30]. The main challenge of this approach has been the limited long‐term survival of the transgenic cells. In a preliminary study of four patients, wound healing was observed in some C7 gene‐corrected grafts; however, responses were variable and declined within 1 year [20]. At 2 years, only 70% of treated wounds remained at least 50% closed, and C7 expression was largely lost [19]. Nevertheless, a successful Phase 3 clinical trial (VIITAL study) was conducted, leading to FDA approval in 2025 for prademagene zamikeracel (Zevaskyn) [31]. A potential advantage of this strategy could be the durable genetic correction of the treated areas; however, this seems not to be the case, as long‐term persistence of therapeutic benefit appears to be variable. Disadvantages of this approach include the invasive nature and the technical complexity of the procedure, which is prone to various challenges, such as contamination, limited cell survival or failure of the transplanted grafts (Table 1). To the best of our knowledge, prademagene zamikeracel is currently available only in selected centers in the USA. Despite these limitations, its development demonstrates that durable restoration of C7 expression and long‐term wound improvement can be achieved through skin grafting [32].

### 2.2 Topical In Vivo Gene Therapy

#### 2.2.1 Background and Mechanism of Action

Beremagene geperpavec (B‐VEC) is a topical gene therapy that delivers two copies of the *COL7A1* coding sequence using a modified herpes simplex virus type 1 (HSV‐1) vector (Figure 1). Following topical application to DEB wounds, the vector infects skin cells, including keratinocytes and fibroblasts. The viral DNA containing *COL7A1* is transported into the cell, where it is transcribed into *COL7A1* mRNA and subsequently translated into C7 protein. The newly synthesized C7 is secreted into the extracellular matrix at the dermal–epidermal junction, where it assembles into anchoring fibrils [16]. These fibrils help restore adhesion between the epidermis and dermis, thereby promoting wound healing in patients with DEB [16, 33]. In a subset of seven patients, biopsies from intact B‐VEC treated skin showed C7 expression and presence of anchoring fibrils at day 15 and 97 [16]. The question of the approximate C7 levels needed for phenotypic improvement and their relevance for partial genetic correction cannot be adequately answered based on the currently available evidence and remains largely speculative. The assumptions are primarily derived from murine studies [11], which have important limitations when extrapolated to the clinical situation in patients with DEB. Findings from genotype–phenotype correlation studies in humans indicate that the amount of C7 required to maintain some skin integrity is much lower and is unlikely to be uniform across different anatomical sites. For example, mechanically stressed areas such as the knees may require substantially higher levels of C7 than less exposed sites such as the abdomen. Furthermore, wound healing and skin stability are influenced by numerous patient‐ and wound‐specific factors, including age, body mass, physical activity, wound chronicity, infection, inflammation, tissue destruction and local mechanical stress.

**FIGURE 1:**
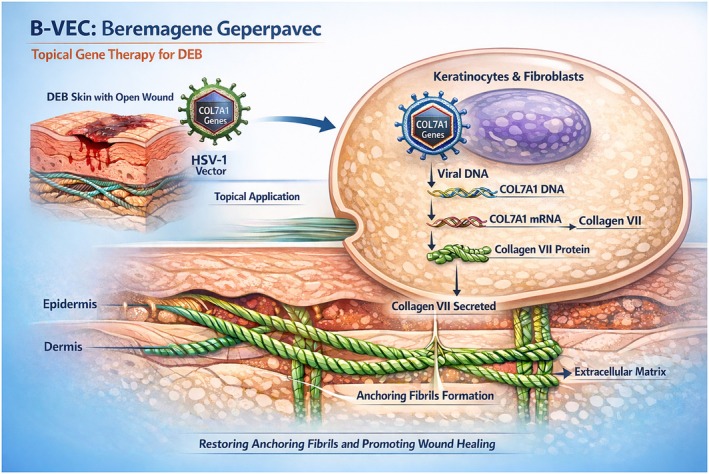
Mechanism of action of B‐VEC (Beremagene geperpavec) in dystrophic epidermolysis bullosa (DEB). B‐VEC is a topical gene therapy designed for treating wounds in patients with DEB. It consists of two copies of the *COL7A1* coding sequence, encapsulated within a modified herpes simplex virus type 1 (HSV‐1) vector. Once inside the cell, the HSV‐1 viral DNA is transcribed and translated into collagen VII, which is then secreted into the extracellular space to facilitate the assembly of anchoring fibrils. Figure created with ChatGPT.

Importantly, the experimental conditions in mouse models differ markedly from the clinical reality. Murine wounds are typically acute, heal rapidly and lack many of the complicating factors observed in patients with EB, such as chronic inflammation, recurrent infection, fibrosis and repeated trauma, all of which may affect the efficiency of B‐VEC‐mediated gene transfer and C7 expression. At present, definitive conclusions regarding the amount of C7 required for durable wound healing in different clinical contexts in humans cannot be drawn.

#### 2.2.2 Clinical Trials and Open‐Label Extension Studies

The phase 1/2 and 3 clinical trials showed good tolerability and safety of B‐VEC. Around 70% of the treated wounds were completely closed after 6 and 3 months of treatment as compared to 20% of the placebo‐treated wounds [16, 33]. Open‐label extension studies were conducted in the USA and Japan [34, 35]. Patient‐reported outcomes indicated high levels of treatment satisfaction but were inconclusive with regard to quality of life. Among rollover subjects, wounds that received B‐VEC during phase 3 maintained high closure rates during the open‐label extension (range 61.1%–89.5%, assessed baseline to month 12) [34].

#### 2.2.3 Real‐World Experiences and Own Preliminary Observations

Only a few publications have reported real‐world experience with B‐VEC, demonstrating its potential. For example, B‐VEC has been successfully used to promote closure of large skin defects following excision of cutaneous squamous cell carcinoma in a patient with recessive DEB (RDEB) [36]. In addition, a recent case report described the successful off‐label use of a temporarily increased B‐VEC dose in an RDEB patient with extensive wounds, suggesting that higher dose volumes may be beneficial for patients with a high wound burden [37]. A RDEB patient with cicatrizing conjunctivitis in both eyes received ophthalmic administration of B‐VEC, which was associated with improved visual acuity after surgery [38]. Finally, we reported on the benefit of early B‐VEC administration in an infant with RDEB [39].

Starting in January 2024, we enrolled eight patients with severe RDEB and one patient with intermediate RDEB in the early access program (ages ranging from 9 months to 43 years, 5 females). The duration of treatment ranged from 1 to 12 months (median: 6 months) (*submitted manuscript*). B‐VEC was applied weekly to all wounds and blisters according to the FDA‐approved label. Here, we report preliminary data from our real‐world experience with this treatment.

Overall, patients tolerated the therapy well and reported no pain or treatment‐related adverse effects. Chronic wounds generally required a greater number of treatment sessions to achieve closure, whereas acute wounds typically healed within 7–14 days with minimal or no visible scarring (Figure 2). While most healed wounds remained closed, recurrences were common in areas exposed to high mechanical friction (e.g., head, legs and arms) or in pruritic regions prone to scratching. Additional factors, such as pre‐existing scarring, chronic inflammation and repeated mechanical trauma from scratching, may influence wound healing outcomes. Regarding adherence to the recommended weekly treatment schedule, some doses were missed due to holidays, unrelated illnesses or logistical difficulties in travelling to the treatment center. However, seven patients were able to continue treatment at home after their wound care teams received appropriate training.

**FIGURE 2:**
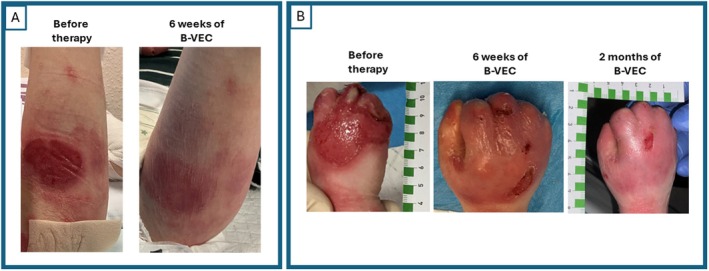
Healing of wounds in a 5‐year‐old patient with severe recessive dystrophic epidermolysis bullosa treated with B‐VEC: Acute wounds (Panel A) healed within weeks with minimal or no visible scarring, whereas chronic wounds required more treatment sessions to achieve wound closure (Panel B).

Our preliminary data from the early access program demonstrate that B‐VEC is a well‐tolerated therapy and shows promising results in reducing wound area in this case series. Chronic wounds—particularly those occurring on a background of scarred skin—often require longer treatment durations, whereas acute wounds tend to heal rapidly and remain closed. Patient compliance, overall clinical condition, pre‐existing scarring and wound manipulation may all affect treatment outcomes. To improve adherence, caregivers should be trained to administer the treatment at home, thereby reducing the burden of travel for patients. Further research is needed to better understand the pathogenic factors involved in chronic wounds and to refine strategies for maximizing the therapeutic benefits of B‐VEC.

#### 2.2.4 Practical Considerations of B‐VEC Treatment

B‐VEC treatment proved easy to administer and was generally well tolerated in our cohort. Importantly, we did not observe treatment‐related pain, which represents a major advantage in this patient population. In most treated wounds, we observed a progressive reduction in wound area and depth over time. Systematic photographic documentation of wounds and previously affected areas during follow‐up was particularly helpful to monitor treatment response and to support clinical decision‐making.

B‐VEC should be consequently applied weekly to clean wounds free of crusts or biofilm until complete closure (Figure 3) [40]. Prior to application, wounds are cleaned with sterile saline, gently dried and the surrounding margins are protected with a thin layer of Vaseline. Under treatment, the area and depth of the wounds typically decrease, and the skin gradually gains stability. Healed areas should be protected from friction until the skin is fully stabilized. Vaseline or other wound ointments (e.g., panthenol) can be used for this purpose. A major advantage of B‐VEC therapy is the feasibility of home administration, which is particularly beneficial for patients with a high disease burden. Coordinated involvement of the dispensing pharmacy, the wound management team, patient support programs and the EB center further enhances wound control, treatment adherence and overall clinical outcomes.

**FIGURE 3:**
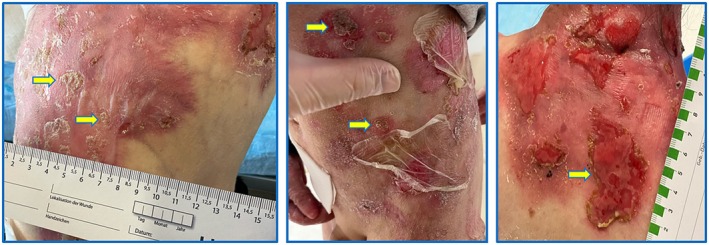
Representative examples of wounds unsuitable for B‐VEC treatment (indicated by the yellow arrows). Wounds exhibiting crusts, biofilm or signs of infection should be managed and stabilized prior to application of B‐VEC. Effective wound preparation—including gentle cleaning, removal of crusts and control of infection—is essential to maximize gene therapy efficacy and ensure proper transgene delivery and healing.

In infants and children, early treatment initiation appeared particularly beneficial, not only with regard to wound healing but also in terms of overall well‐being and physical development, likely due to reduced pain, inflammation and chronic wound burden [39]. In contrast, in adult patients, especially those with long‐standing disease, healing of chronic wounds located in heavily inflamed or scarred areas remained more challenging and often required longer treatment periods.

Several factors that are known to interfere with wound healing should be carefully considered when evaluating treatment response. These include bacterial colonization and superinfection, pre‐existing or newly diagnosed squamous cell carcinoma, pruritus with consecutive wound manipulation, severe anaemia, malnutrition, advanced tissue destruction and poor general clinical condition (Figure 4). Careful management of these factors appears essential to optimize therapeutic outcomes.

**FIGURE 4:**
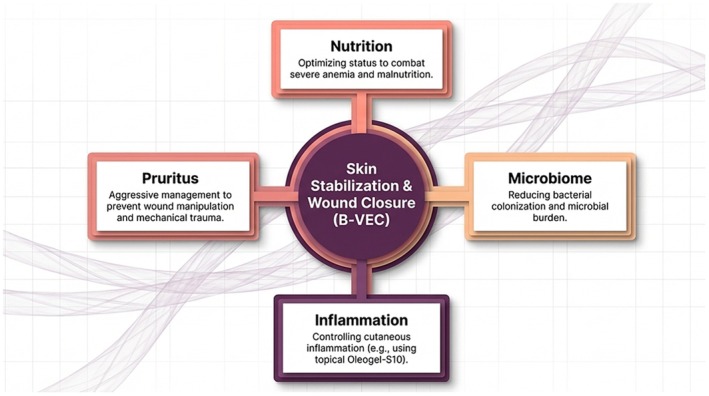
Key factors supporting wound healing and skin stabilization in dystrophic epidermolysis bullosa. A multimodal therapeutic approach is required and includes B‐VEC therapy, optimization of nutritional status, reduction of pruritus, control of cutaneous inflammation with topical anti‐inflammatory treatments (e.g., Oleogel‐S10), optimized skin care and reduction of microbial burden to promote wound healing and improve skin stability. Figure created with NotebookLM.

## 3 Ongoing Clinical Trials

### 3.1 Genetically‐Corrected Fibroblasts

Fibroblasts have been considered for the treatment of DEB wounds because they are easy to culture and because of their putative role in the pathophysiology of DEB (Figure 5) [41]. Several studies investigated their therapeutic potential in the DEB mouse model and in humans [5, 11, 42]. The major limitation of allogeneic fibroblasts is the low efficacy due to the short survival of these cells along with the low amount of generated C7 [43]. In a single‐center, open‐label Phase I trial, four adults with RDEB received three intradermal injections (~1 × 10^6^ cells/cm^2^ of intact skin) of *COL7A1*‐modified autologous fibroblasts and were followed up for 12 months. The treatment was well tolerated and demonstrated potential efficacy, as evidenced by the presence of *COL7A1* transgene and subsequent C7 restoration in vivo within the treated skin at 1 year post‐therapy [42]. A Phase 1/2 clinical study (D‐Fi, NCT02810951) evaluated *COL7A1*‐modified autologous fibroblasts in six patients with RDEB. In this study, 80% (8/10) of treated chronic wounds demonstrated at least 90% closure 12 weeks after the first D‐Fi injection, whereas none of the untreated wounds healed. The treatment was well tolerated, with only occasional reports of transient redness or discoloration at the injection site. Two Phase 3 clinical trials (NCT04213261, NCT06892639) evaluating the efficacy of autologous gene‐corrected fibroblasts for DEB wound healing are currently ongoing.

**FIGURE 5:**
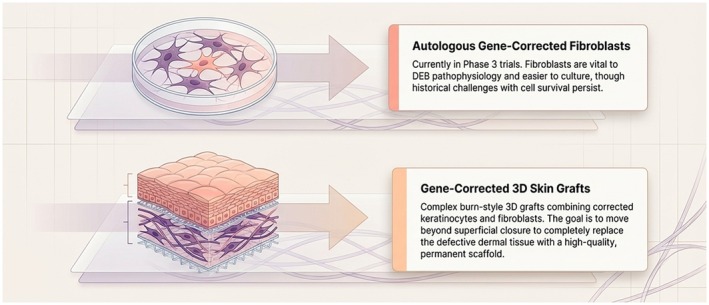
Schematic representation of therapeutic strategies aimed at restoring dermal integrity in DEB. Patient‐derived fibroblasts are genetically corrected ex vivo (e.g., via retroviral or lentiviral *COL7A1* transduction) and expanded for intradermal injection to promote local C7 expression and dermal repair. A more comprehensive approach involves generating three‐dimensional skin grafts composed of autologous, gene‐corrected keratinocytes and fibroblasts, which replace both epidermis and dermis. These engineered grafts are transplanted onto affected skin areas to restore anchoring fibrils, improve dermal–epidermal adhesion and enhance long‐term skin stability. Both strategies represent personalized, cell‐based gene therapies for the durable correction of DEB skin fragility. Figure created with NotebookLM.

### 3.2 Gene‐Corrected Skin Grafts

Another clinical trial (NCT01263379) is currently evaluating the efficacy and safety of grafting genetically corrected keratinocytes and fibroblasts assembled into three‐dimensional skin grafts, a strategy previously applied in the treatment of burns (Figure 5) [44]. This approach is complex and resides the risks related to cell survival in culture and after grafting. A potential advantage is theeplacement of pathologic DEB dermal tissue with genetically modified skin, which may improve the structural and functional quality of the grafts.

A similar strategy, combining gene therapy and tissue‐engineered skin substitutes for potential permanent closure of DEB wounds, has been developed at the LOEX research center in Quebec City [45, 46]. A Phase 1/2 clinical trial (GMEB‐SASS, NCT07193134) is currently underway, employing a retroviral vector to introduce a corrected *COL7A1* gene into genetically modified skin grafts for the treatment of RDEB.

## 4 Natural Gene Therapy in DEB

Revertant mosaicism, a form of natural gene therapy, has been described in all types of DEB, occurring in both keratinocytes and fibroblasts [47, 48, 49, 50, 51].

Revertant areas have been utilized to treat wounds, either via punch grafts or cultured epidermal autografts. In one study, eight wounds of three RDEB patients were treated using cultured autografts. Wound epithelization rates per patient at 4 weeks were 87.7%, 100% and 57.0%, respectively, and the effects persisted for at least 76 weeks post‐ transplantation. Although revertant mosaicism offers potential as a source of autologous cell therapies, several limitations exist: the variable presence and proportion of revertant cells, difficulties in expanding revertant keratinocytes while preserving their genotype, as well as technical challenges requiring specialized expertise for detecting revertant mosaicism, isolating revertant tissue and producing durable grafts (Table 1).

## 5 Perspectives

Despite the inherent limitations and ongoing challenges, the recent successes in gene therapy represent a major milestone in the treatment of DEB. These advances have provided invaluable lessons regarding vector design, cell sourcing, grafting strategies and clinical management, while driving the evolution of increasingly sophisticated technologies.

A qualitative comparison of ‘typical efficacy ranges’ in wound healing across the different gene and cell therapy approaches, based on the available published data, is not scientifically justified and would represent an artificial endpoint, given the marked diversity of wounds and study designs (Table 1). Wound healing in RDEB is highly heterogeneous, with healing trajectories varying substantially according to anatomical location, wound size, age, chronicity, local mechanical stress and patient‐specific factors. Direct comparisons of treatments across such diverse wounds are therefore of limited interpretability and may lead to misleading conclusions. Moreover, wound closure alone does not adequately capture the clinical benefit of a corrective treatment in RDEB. Clinically more relevant endpoints include improved skin stability, reduction of the overall wound burden, restoration of function/mobility, decreased pain and mitigation of systemic inflammation and other disease‐related complications. These outcomes better reflect the multidimensional therapeutic impact of treatment in this complex multisystem disorder.

**TABLE 1:** Overview of gene therapies for DEB, approved or in a clinical stage of development.

Therapy/Characteristics	Gene corrected keratinocyte grafts	Topical in vivo gene therapy	Gene corrected fibroblasts	Gene corrected dermal‐epidermal grafts	Revertant grafts
Route of administration	Autologous, cell‐based gene therapy (skin graft) One‐time surgical application under anaesthesia	Redosable, topical gene therapy (gel) Weekly topical application in a grid pattern	Local intradermal injections into the wounds Three or more treatment sessions.	Grafting One‐time surgical application	Grafting One‐time surgical application
Phase of development	Approved in the USA	Approved in USA; Europe and Japan	Phase 1/2 Phase 3 ongoing	Phase 1/2 ongoing	Compassionate use in three patients
Major efficacy outcomes	At 6 months, 81% of the treated wounds were at least 50% healed from baseline for prademagene zamikeracel vs. 16% of control wounds.	At 6 and 3 months, about 70% of the B‐VEC treated wounds achieved complete wound healing vs. 20% with placebo	At 3 months 80% of treated chronic wounds demonstrated at least 90% closure.	NA	Variable wound reepithelization rates (57%–100%), persisted 76 weeks post‐ transplantation
Main limitations/safety considerations	Availability of the technology (specialized infrastructure, manufacturing capacity, cost and access) Risks associated with ex vivo expansion of patient‐derived cells (contamination, genomic instability, variable cell quality, manufacturing complexity and treatment delays) At least 5–10 days of immobilization (often hospitalized) Limited durability	No severe adverse events Limited durability	Availability of the technology (specialized infrastructure, manufacturing capacity, cost and access) Risks associated with ex vivo expansion of patient‐derived cells (contamination, genomic instability, variable cell quality, manufacturing complexity and treatment delays) No severe adverse events Limited durability	Availability of the technology (specialized infrastructure, manufacturing capacity, cost and access) ‐Risks associated with ex vivo expansion of patient‐derived cells (contamination, genomic instability, variable cell quality, manufacturing complexity and treatment delays)—Immobilization (often hospitalized)	Revertant mosaicism is not present in all DEB patients Risks associated with ex vivo expansion of patient‐derived revertant cells (contamination, genomic instability, variable cell quality, manufacturing complexity and treatment delays)
Main advantage	Durability in some cases	Home administration Noninvasive			

Key questions remain, including how to optimally combine different therapeutic approaches, how to stratify patients based on genotype, disease severity or wound characteristics, and how to maximize long‐term efficacy and durability. Equally critical is ensuring broad accessibility, both in terms of geographic availability and cost, to translate these breakthroughs into meaningful improvements for the wider DEB patient community. As research continues, integrating gene‐, cell‐ and tissue‐based therapies with supportive care and personalized strategies will likely define the next frontier in achieving durable skin stability and improved quality of life for patients.

Future treatment algorithms may position topical gene therapy (B‐VEC) as a backbone therapy for acute and subacute wounds, while reserving graft‐based or fibroblast‐based approaches for highly fibrotic, therapy‐refractory chronic lesions. Such strategies will likely be most effective when integrated into a multimodal treatment approach that also addresses nutritional status, control of inflammation and infection, optimization of the wound microbiome, pruritus management and other supportive measures to maximize wound healing and improve overall patient outcomes. Further development of gene therapies for mucosal involvement represents an important future direction, including formulations and delivery strategies targeting the ocular surface [38], oral cavity and oesophageal mucosa. Effective treatment of these sites could address major sources of morbidity in patients with DEB, improving vision, nutrition, swallowing function and overall quality of life.

## Author Contributions

Cristina Has: conceptualization, methodology, writing – original draft, writing – review and editing, project administration, data curation, investigation and formal analysis. Meropi Karakioulaki: formal analysis, investigation, visualization, data curation, writing – original draft and writing – review and editing.

## Conflicts of Interest

C.H. has served as an advisor for Chiesi, Krystal, Leo Pharma and Almirall and as a speaker for Chiesi and Krystal.

## Data Availability

The data that support the findings of this study are available on request from the corresponding author. The data are not publicly available due to privacy or ethical restrictions.
